# Theaflavin Reduces Oxidative Stress and Apoptosis in Oxidized Protein-Induced Granulosa Cells and Improves Production Performance in Laying Hens

**DOI:** 10.3390/ani15060845

**Published:** 2025-03-15

**Authors:** Ling Zhou, Li Lv, Pinyao Zhao, Jinwei Zhang, Yan Liu, Wei Zhao, Keying Zhang, Shuwen Du

**Affiliations:** 1 Department of Quality Management and Inspection & Quarantine, Yibin University, Yibin 644001, China; zhouling@yibinu.edu.cn (L.Z.); zhaopy@yibinu.edu.cn (P.Z.); zhangjinwei@yibinu.edu.cn (J.Z.);; 2 Institute of Brain Science and Diseases, West China Hospital of Sichuan University, Chengdu 610041, China; lvlisunny2012@163.com; 3 Institute of Animal Nutrition, Key Laboratory for Animal Disease-Resistance Nutrition of China Ministry of Education, Sichuan Agricultural University, Chengdu 611134, China; zkeying@sicau.edu.cn

**Keywords:** theaflavin, oxidized corn gluten meal, granulosa cells, production performance, laying hens

## Abstract

This study aims to investigate the effects of theaflavins on production performance and egg quality in laying hens fed oxidized corn gluten meal while evaluating their antioxidant and anti-apoptotic effects on granulosa cells (GCs) from chicken follicles. In total, 600 Lohmann commercial laying hens, aged 64 weeks, were randomly assigned to four treatment groups: a control group, a theaflavin-supplemented group, an oxidized corn gluten meal group, and a combination group. After 8 weeks of feeding, production performance, egg quality, and antioxidant status, along with GC apoptosis and the antioxidant capacity of eggs, were measured. The results demonstrated that oxidized corn gluten meal significantly reduced production performance, antioxidant capacity, and egg quality in laying hens while increasing GC apoptosis. Theaflavin significantly enhanced egg production during weeks 5–8, along with superoxide dismutase activity in the liver, serum, and ovary, alongside egg white reducing power and egg yolk threonine content (*p* < 0.05). Additionally, theaflavin decreased feed conversion ratios during weeks 5–8 and 1–8, lowered egg white malondialdehyde content (*p* < 0.05), and inhibited GC apoptosis. In conclusion, oxidized protein reduced production performance, while theaflavin supplementation partially alleviated its adverse effects.

## Simple Summary

Proteins undergo inevitable oxidization during feed storage, leading to the formation of protein carbonyls and the advanced oxidation of protein products. When laying hens consume oxidized proteins, oxidation stress can be induced, negatively affecting their production performance. Theaflavins, natural antioxidants extracted from black tea, have not been previously investigated for their potential to mitigate the adverse effects of oxidized proteins in laying hens. Therefore, this study aims to investigate whether theaflavins can mitigate the negative effects of oxidized corn gluten meal in feed and improve laying hen performance. The results demonstrated that theaflavins enhance the antioxidant capacity of laying hens, mitigate oxidative stress, and reduce apoptosis in ovarian granulosa cells, ultimately improving egg production rates. These findings suggest that theaflavins represent a promising dietary strategy for mitigating the adverse effects of oxidized corn gluten meal in poultry feed.

## 1. Introduction

Eggs, rich in essential nutrients, including proteins, lipids, vitamins, and minerals, along with embryo development factors, are a staple in daily nutrition [1]. Follicular development, a crucial process, directly influences egg quality and production [2]. Ovarian follicles, serving as the functional units of the ovary, are composed of theca cells, granulosa cells (GCs), and oocytes, which collectively contribute to reproductive function [3]. GCs, a crucial somatic component surrounding the ovary, provide both nutritional and structural support. Consequently, the proliferation, apoptosis, and differentiation of GCs directly affect the fate of follicular development and ultimately determine egg quality.

Reactive oxygen species (ROS), highly reactive molecules generated during oxidative stress, have been extensively investigated. While low concentrations of ROS are essential for biological functions in cells, including GCs, excessive ROS levels can impair cellular function or induce apoptosis. Under oxidative stress induced by advanced oxidation protein products (AOPPs), the ROS-c-Jun N-terminal kinase (JNK)/p38 mitogen-activated protein kinase-p21 pathway is activated, leading toG1/G0-phase arrest in KGN cells [4]. Oxidized proteins are unavoidable during storage and preparation before feeding. After feeding laying hens oxidized protein, genes related to antioxidation in GCs, such as forkhead box O1 (FoxO1), extracellular signal-regulated kinase (ERK), and c-JNK, undergo transcriptional changes, indicating that AOPPs have a significant effect on the fate of GCs [5]. Therefore, eliminating or preventing the detrimental effects of oxidation stress on GCs is crucial for maintaining egg production. Puerarin, a phytoestrogen, has been investigated as a feed additive for laying hens and has been shown to increase egg production and quality by improving antioxidant activity and gut health [6]. The inclusion of essential oils in the diet can improve egg quality by increasing total antioxidant capacity (T-AOC) and superoxide dismutase (SOD) levels in late-laying hens [1]. Quercetin, a natural extract, can mitigate CuSO4-induced toxicity in GCs by scavenging ROS and improving mitochondrial function [7].

Theaflavins, a group of tea polyphenols extracted from black tea fermentation, are important natural antioxidant compounds. These include theaflavin (TF1), theaflavin-3-gallate (TF2A), theaflavin-3′-gallate (TF2B), and theaflavin-3,3′-digallate (TF3). Among these, TF3 has been reported to inhibit apoptosis in human GC apoptosis and improve their antioxidative capacity [8]. Li et al. (2019) reported that theaflavin reduces cerebral ischemia–reperfusion injury by activating the Nrf2 signaling pathway and inhibiting oxidative stress [9]. Research in mice has shown that theaflavin protects against renal ischemia–reperfusion injury by promoting p53 nuclear translocation, activating the p53/GPx-1 pathway and upregulating GPx-1 expression to mitigate oxidative stress and apoptosis [10]. However, to our knowledge, the antioxidant and anti-apoptotic effects of theaflavin on GCs in laying hens and their potential influence on egg quality have not been reported. Therefore, this study aims to investigate the protective and enhancing effects of theaflavin on production performance, egg quality, and antioxidant status in laying hens. Oxidized corn gluten meal was prepared as a source of AOPPs and fed to the hens. Subsequently, GCs were isolated and cultured under AOPP-induced oxidative stress. The antioxidant and anti-apoptotic effects of theaflavin were measured using biochemical assays and immunostaining techniques. Theaflavin as an additive could significantly improve egg quality under AOPP-induced stress, with its protective mechanism potentially relying on antioxidation and anti-apoptotic effects.

## 2. Materials and Methods

### 2.1. Theaflavins and Oxidized Corn Gluten Meal Preparation

TFs were purchased from Dannisi Technology Co., Ltd. (Xian, China), with a purity of 62.9%. The composition included 40.67% TF1, 8.91% TF3, 7.14% TF2A, and 6.18% TF2B.

Oxidized corn gluten meal was prepared by heating fresh corn gluten meal (EPPEN Biotechnology Co., Ltd., Ningxia, China) at 100 °C for 24 h following a previously established method (Table 1) [5].

### 2.2. Birds, Experimental Design, Diets, and Materials

The study was conducted using a 2 × 2 factorial design, with two primary factors: corn gluten meal type (fresh or oxidized) and theaflavin supplementation (0 or 500 mg/kg). The four experimental groups were as follows: 1. the control group (CON) fed a diet containing 10% fresh corn gluten meal; 2. TF2 fed a diet containing 10% fresh corn gluten meal with 500 mg/kg of theaflavin; 3. The oxidized corn gluten meal group (OX) fed a diet containing 10% oxidized corn gluten meal; and 4. the combination group (CB)fed a diet containing 10% oxidized corn gluten meal with 500 mg/kg of theaflavin. Overall, 600 healthy 64-week-old Lohmann commercial laying hens were randomly assigned to four groups (5 hens per cage, 15 hens per replicate, and 10 replicates per group). The experiment started at week 64 and continued for 8 weeks. The diets were formulated following the Agricultural Trade Standards of China [11]. Table 2 presents the feed components and nutrient profiles.

### 2.3. Animal Care

The experiment was conducted at Yibin Shangougou Agricultural Science and Technology Co., Ltd. (Yibin, China). All chickens were housed in cages, with five hens per cage (three cages per replicate), under a 16 h light and 8 h dark cycle (16L:8D). Each cage, measuring 60 × 60.3 × 70 cm, was equipped with an individual feeder and water supply. Feed and water were supplied ad libitum. The temperature and relative humidity were maintained at 20 ± 4 °C and 50–65%, respectively. Eggs were collected and counted daily at 9:00 a.m.

### 2.4. Determination of Parameters

#### 2.4.1. Determination of Corn Gluten Meal Parameters

Fresh and oxidized corn gluten meal: The dry matter, crude protein, crude fat, protein carbonyl, free sulfhydryl, and total disulfide–sulfhydryl contents were measured using specific assay kits from Nanjing Jiancheng Bioengineering Institute (Nanjing, Jiangsu Province, China) with a Multiskan Spectrum Reader (Model 1500; Thermo Fisher Scientific, Nyon, Switzerland).

#### 2.4.2. Laying Hen Performance

The weight and number of eggs laid were recorded daily, using each replicate as a unit. Additionally, dirty and broken eggs were recorded. The egg production rate, feed conversion ratio (FCR), average egg weight, and average daily feed intake (ADFI) were calculated weekly following to the method used by Zhou et al. (2020) [5].

#### 2.4.3. Antioxidant Status in Laying Hens

At the end of the 8th week, one chicken was randomly selected from each replicate for serum collection. Overall, 15 mL of blood was drawn from the wing vein into a tube without anticoagulant. The blood samples were centrifuged at 3500 rpm for 10 min, while the resulting serum was stored at −20 °C for subsequent antioxidant analysis. After blood collection, the liver and ovary of each laying hen were harvested and immediately stored at −80 °C for subsequent analysis. The liver and ovarian tissue samples were homogenized in PBS and centrifuged at 4000 rpm for 10 min to obtain the supernatant. The T-AOC, SOD, GSH-Px activity, and malondialdehyde (MDA) content in the liver, ovarian tissue, and serum were analyzed using reagent kits (Jiancheng Bioengineering Institute, Jiangsu, China).

#### 2.4.4. Granulosa Cell Apoptosis In Vitro

Isolation and culture of granulosa cells: Primary GCs were isolated following previously described methods (Gilbert et al., 1977; Lin et al., 2025) [12,13]. Briefly, small yellow hierarchical follicles were collected, and the granulosa layers were sterilized before being cut into tissue fragments. Subsequently, the fragments were digested in a 1 mg/mL type II collagenase solution (BaiTai Biotechnology, Chengdu, China) for 5–10 min. The resulting cell suspension was filtered through a 200-mesh cell sieve. The cells were seeded in Dulbecco’s Modified Eagle Medium (DMEM), supplemented with 10% fetal bovine serum (Gibco, Grand Island, NY, USA) and 0.1% penicillin-streptomycin (Invitrogen, Carlsbad, CA, USA). Subsequently, the GCs were cultured at 37 °C in a humidified incubator with 5% CO_2_ and 95% air. All experiments were conducted with at least three biological replicates.

Cell Counting Kit-8 assay for cell proliferation and cytotoxicity: The method for measuring GC proliferation and cytotoxicity with the CCK8 kit was carried out in accordance to Lin et al. (2022) [13]. Briefly, GCs from each treatment group were seeded in 96-well culture plates at a density of 2000 cells per 100 µL per well for the proliferation assay or 5000 cells per 100 µL for the cytotoxicity assay. Subsequently, 10 µL of the Cell Counting Kit-8 (CCK-8) solution (Super-Enhanced Cell Counting Kit-8, Lot No.: C0048S, Beyotime, Shanghai, China) was added to each well. After a 1 h incubation at room temperature, the absorbance was measured at 450 nm using a microplate reader. DMEM was used for the in vitro cell-based assays. A 50 µM concentration of theaflavin (Catalog No.: B20143; Yuanye, Shanghai, China) was used in subsequent cellular assays.

Ki67 immunostaining in granulosa cells: The Ki-67 immunostaining was applied to assess the ability of theaflavin to promote GCs’ proliferation based Urata et al. (2023) [14]. Briefly, GCs were plated at 2.5 × 10^5^ cells per well and incubated for 24 h. After incubation, AOPPs (KTB1060) and AOPPs with theaflavin were added, followed by an additional 4 h of incubation. Subsequently, the cells were fixed with 4% paraformaldehyde fixation solution (P0099, 100 mL, Beyotime) for 10 min. After fixation, the cells were rinsed with PBS and imaged using a fluorescence microscope.

Flow cytometric analysis of granulosa cell apoptosis: The effect of theaflavin on the apoptosis of chicken GCs was assessed using flow cytometry with propidium iodide (PI) and annexin V-fluorescein isothiocyanate (FITC) labeling according to Wang et al. (2021) [15]. GCs were cultured at 1 × 10^6^ cells per well for 24 h. The culture medium was replaced with Annexin V Binding Buffer at a final concentration of 1 × 10^5^ cells per mL. A 300 µL aliquot of the cell suspension was transferred to an Eppendorf (EP) tube, followed by the addition of 3 µL of Annexin V-FITC and 3 µL of propidium iodide solution (C10625, Beyotime). The mixture was incubated for 20 min in the dark, and flow cytometry was performed for sample analysis.

#### 2.4.5. Assessment of Egg Quality

Egg quality was determined on the final day of the 8th week by randomly collecting four eggs per replicate (*n* = 40). The parameters analyzed included egg weight (EW), eggshell color (EC), eggshell strength (ESS), eggshell thickness (ET), Haugh unit (HU), yolk height (YH), and yolk color (YC). EC—measured as L* (brightness), a* (redness), and b* (yellowness)— was evaluated using a Chroma Meter CR-410 (Konica Minolta, Tokyo, Japan). ESS was determined using Eggshell Strength Tester Model-II (Robotmation, Tokyo, Japan). ET was calculated as the mean of measurements taken from the top, middle, and bottom of the eggshell using a Vernier caliper. YH, YC, and HU were determined using an EMT-7300 Multi-Function Egg Tester (Robotmation, Tokyo, Japan). The yolk index was calculated using the following formula: yolk index = yolk length/yolk width at week 8. Vitelline membrane strength was determined using a texture analyzer.

#### 2.4.6. Evaluation of Antioxidant Capacity in Eggs

The egg yolk and white were separated and homogenized using four eggs per replicate at week 8. The antioxidant status (*n* = 10) was assessed by measuring 2,2-Diphenyl-1-Picrylhydrazyl (DPPH) radical scavenging activity in both the egg yolk and egg white, alongside reducing power, MDA levels, and protein carbonyl content.

**DPPH radical scavenging activity:** The DPPH value of the samples was measured using a modified method based on Shang et al. [16]. Briefly, 1 mL of egg yolk (10 mg/mL) or 1 mL of lyophilized egg white (50 mg/mL) was mixed with 1 mL of a 95% ethanol solution of DPPH. The mixture was incubated at room temperature for 30 min, after which absorbance was measured at 517 nm against a blank solution as the reference. The DPPH radical scavenging activity (%) was calculated using the following formula: [(Absorbance of Blank − Absorbance of Sample)/Absorbance of Blank] × 100.

**Measurement of reducing power:** The method for determining reducing power was modified based on the method described by Hamdani et al. [17]. A 0.5 mL suspension of ground egg white and yolk (40 mg yolk/mL or 200 mg freeze-dried egg white/mL) was mixed with 0.5 mL of PBS buffer (pH 6.6) and 2.5 mL of 1% potassium ferricyanide (K_3_[Fe(CN)_6_]) before being incubated at 50 °C for 20 min. Subsequently, the solution was mixed with an equal volume of 10% trichloroacetic acid and centrifuged at 3000 rpm for 10 min. A 0.5 mL aliquot of the supernatant was mixed with 0.5 mL of distilled water and 0.1 mL of 0.1% ferric chloride, and absorbance was measured at 700 nm using spectrophotometry. A higher absorbance value indicated greater reducing power.

**MDA and protein carbonyl content:** The MDA and protein carbonyl content in egg yolk and white were measured using a chemical colorimetric method with specific detection kits (Jiancheng Bioengineering Institute, Jiangsu, China). All assays were conducted and interpreted strictly following the manual instructions.

#### 2.4.7. Free Amino Acid Detection

The detection method for free amino acids in eggs was modified according to a previous study [18]. Briefly, 100 mg of frozen yolk was ground with 1 mL of 5-sulphosalicylic acid (10%) and centrifuged at 12,000 rpm for 10 min. The supernatant was filtered through a 0.22 µm filter and analyzed using an amino acid analyzer (L-8900, Hitachi, Tokyo, Japan).

### 2.5. Statistical Analysis

For egg quality and production parameter analysis, a 2 × 2 factorial analysis in the GLM program (IBM SPSS Statistics for Windows, Version 23.0, IBM Corp., Armonk, NY, USA) was used, with theaflavin, oxidized corn gluten meal, and their interaction as main factors, and each replication as a unit. Turkey’s test was used to determine any significant interactions. For in vitro assays, a one-way analysis of variance was used. * *p* < 0.05, ** *p* < 0.01, *** *p* < 0.001, and “ns” indicates no statistical significance.

## 3. Results

### 3.1. Effect of Theaflavin and Oxidized Corn Gluten Meal on the Production Performance of Laying Hens

Compared to the CON group (−OX, −TF), the laying rate in the oxidized corn gluten meal group (+OX, −TF) decreased significantly from week 5 (*p* < 0.05, Table 3). Theaflavin supplementation improved egg production compared to the OX group, indicating its protective potential. No significant difference in ADFI (*p* > 0.05) was observed among TF treatments. However, the OX-treated hens had significantly lower ADFI. The FCR significantly decreased (*p* < 0.05) in groups supplemented with theaflavin. Egg weight, dirty egg percentage, and broken egg percentage did not differ among the treatment groups. These findings suggest that theaflavin supplementation can mitigate the adverse effects of oxidized corn protein and improve the productivity of laying hens.

### 3.2. Effect of Theaflavin and Oxidized Corn Gluten Meal on the Antioxidant Status of Laying Hens

Theaflavin supplementation significantly increased SOD levels in the liver, serum, and ovary (*p* < 0.05, Table 4). Oxidized corn gluten meal significantly (*p* < 0.05) decreased serum SOD, ovary T-AOC, and GSH-Px while significantly increasing liver MDA content. A significant theaflavin × oxidized corn gluten meal interaction was observed for serum T-AOC (*p* < 0.05), with the CT group showing higher serum T-AOC levels compared with the OX group. These results suggest that theaflavin has antioxidant potential.

### 3.3. Apoptosis of Granulosa Cell In Vitro

#### 3.3.1. AOPP-Induced Apoptosis in Granulosa Cell

**Figure 1:**
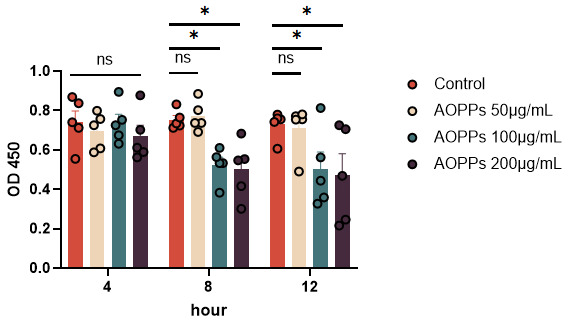
AOPPs decreased GC viability in a dose- and time-dependent manner. * *p* < 0.05; ns, no significance. N = 5 for each group at each time point.

GCs were co-cultured with AOPPs at doses of 50–200 µg/mL for 4–12 h. As seen in Figure 1, higher doses of AOPPs induced more severe apoptosis, while longer exposure reduced cell viability (Figure 1). To explore the protection mechanisms of theaflavin, subsequent assays used the highest AOPP concentration (200 µg/mL).

#### 3.3.2. Theaflavin Attenuated Apoptosis of GCs Induced by AOPPs via Antioxidation

**Figure 2:**
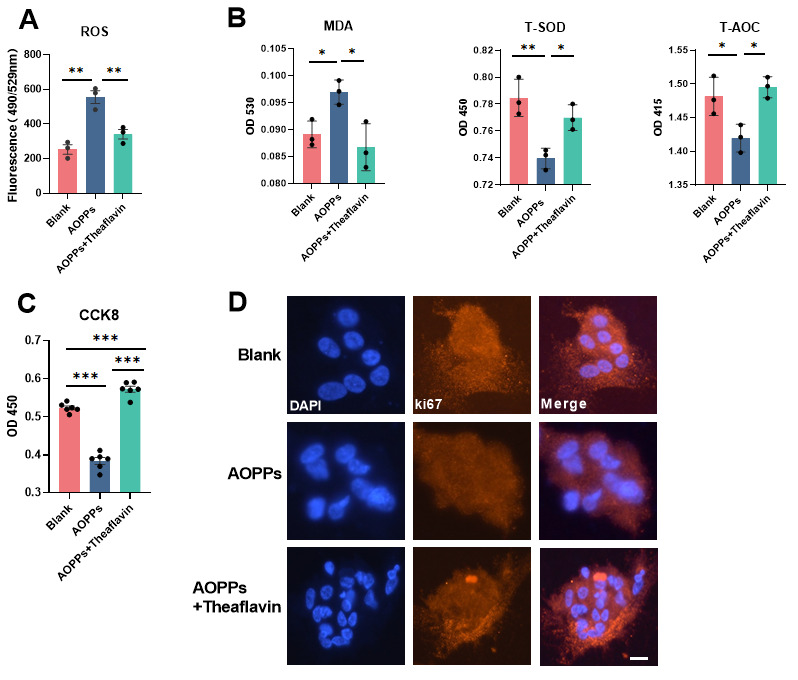
Theaflavin attenuated oxidative stress induced by AOPPs and promoted the proliferation of GCs. (**A**). Theaflavin inhibited ROS generation under AOPP stress (N = 3). (**B**) theaflavin blocked the increase in MDA induced by AOPPs and increased both total SOD and total AOC on GCs under AOPPs (N = 3). (**C**). Theaflavin protected GCs from AOPP oxidant stress and promoted proliferation, as indicated by CCK8 (N = 6). (**D**). Immunostaining of Ki67 was observed, an indicator of proliferation, surrounding the DAPI signal. The bars are 10 µm. One-way ANOVA, * *p* < 0.05, ** *p* < 0.01, *** *p* < 0.001.

Since He et al. [8] reported the antioxidant effects of theaflavin, we evaluated its effect on GCs under AOPP-induced oxidative stress. ROS levels induced by AOPPs and the protective effects of theaflavin were detected using a ROS ELISA kit (Figure 2A,B). GCs co-cultured with theaflavin and AOPPs had significantly lower ROS levels than those treated with AOPPs alone (Figure 2A), indicating that theaflavin inhibits AOPP-induced oxidation. Furthermore, theaflavin treatment reduced MDA levels, a lipid oxidation marker, while increasing antioxidant capacity (T-AOC) and T-SOD, even under AOPP stress. These findings suggest that theaflavin has systemic antioxidant properties.

#### 3.3.3. Theaflavin Promoted Proliferation Under AOPP Stress

GCs have the potential to proliferate. Biochemical and immunostaining assays were used to determine whether theaflavin promotes GC proliferation. The CCK8 signal, an indicator of viable cell count, was significantly higher in the theaflavin group than in the AOPP group (*p* < 0.01, Figure 2C). A similar trend was observed in the immunostaining assay, where Ki67, a proliferation marker, showed greater co-localization with DAPI in the theaflavin group (Figure 2D).

#### 3.3.4. Theaflavin Protected GCs from Early Apoptosis Under AOPP Stress

**Figure 3:**
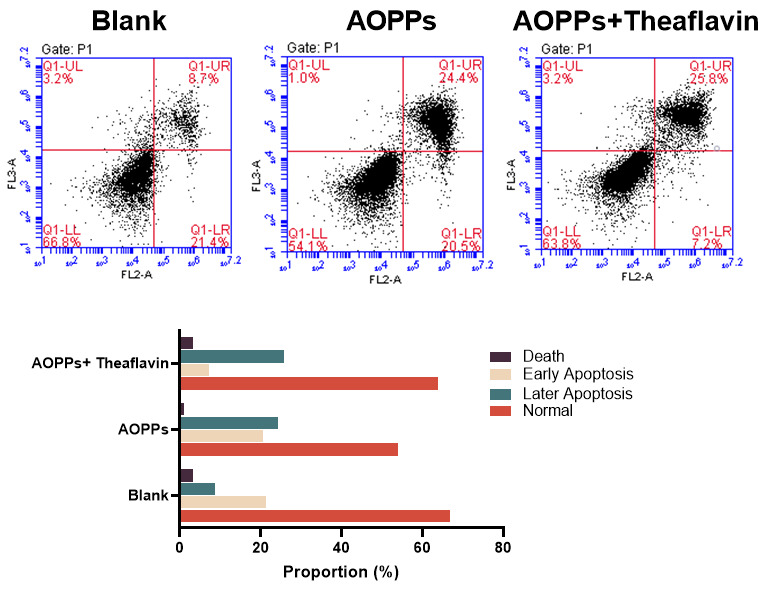
Theaflavin blocked early apoptosis induced by AOPPs on GCs. Upper panel, a representative sample flow cytometry analysis. Lower bar graph, the difference in the proportion of GCs within each group.

To determine the anti-apoptotic effect of theaflavin on GCs, we used an Annexin V-FITC/PI kit with flow cytometry. The number of normal GCs decreased in the AOPP group compared to the blank and AOPP + theaflavin groups (Figure 3). Notably, early apoptosis (FITC high, PI low zone) was lower in the AOPP + theaflavin group than in the AOPP group, indicating that theaflavin enhanced GC resistance to AOPPs. However, the percentage of later apoptosis or necrosis (FITC high, PI high zone) did not differ between the AOPP and AOPP + theaflavin groups, indicating that theaflavin did not affect GCs during the late apoptosis process.

### 3.4. Effect of Theaflavin and Oxidized Corn Gluten Meal on the Egg Quality of Laying Hens

Table 5 presents the effect of oxidized corn gluten meal and theaflavin supplementation on egg quality. After 8 weeks, egg quality parameters showed no significant response to dietary treatment (*p* > 0.05). However, oxidized corn gluten meal significantly decreased yolk color compared to the control group, and theaflavin did not mitigate this effect.

### 3.5. Effect of Theaflavin and Oxidized Corn Gluten Meal on the Antioxidant Capacity of Eggs

Oxidized corn gluten meal significantly decreased the DPPH value and reduced the power of egg yolk (*p* < 0.05, Table 6), while theaflavin did not affect egg yolk antioxidation. A significant theaflavin and oxidized corn gluten meal interaction was observed for the DPPH value (*p* < 0.05), with the CON and TF groups exhibiting higher DPPH values than the other groups. In contrast, theaflavin enhanced the reducing power and decreased the MDA content (*p* < 0.05) in egg whites, demonstrating its antioxidant effect.

### 3.6. Effect of Theaflavin and Oxidized Corn Gluten Meal on Free Amino Acids in Eggs

Oxidized corn gluten meal significantly decreased aspartate, tryptophan, and arginine levels in egg yolk (*p* < 0.05; Table 7), while theaflavin significantly increased threonine content *p* < 0.05). A significant theaflavin and oxidized corn gluten meal interaction was observed for alanine, methionine, tyrosine, and arginine (*p* < 0.05). The OX and TF groups had higher alanine levels than the CON group, while the OX and TF groups had higher methionine levels than the CON group. The TF group had higher tyrosine levels than the OX and CB groups and higher arginine levels than all other groups (*p* < 0.05).

## 4. Discussion

Several studies show that feeding laying hens with oxidized protein significantly decreases productivity [19] and alters the transcription of FoxO1, extracellular ERK, and c-JNK [5]. As a source of AOPPs, oxidized corn gluten meal may influence laying performance and egg quality. In this study, hens were fed 10% oxidized corn gluten meal for 8 weeks, resulting in a 1.2% decrease in egg production and significant increases in ADFI (1%) and FCR (2.9%). Our results align with our previous study [5].

During the heating of corn gluten meal, protein oxidation produces AOPPs and protein carbonyls, resulting in various oxidant levels in feed [20,21,22]. Excess oxidant intake can overwhelm the cellular antioxidant defense system, causing oxidative stress [23]. A previous study reported that oxidized corn gluten meal decreases the antioxidant status of laying hens [5], consistent with our findings that it decreased serum SOD, ovary SOD, and GSH-Px, while increasing liver MDA content, indicating oxidative stress and its detrimental effects. Theaflavins, the main antioxidant compounds in black tea, improved antioxidant status in cell cultures and animal models [24]. This effect on laying hens suggests that they may partially counteract the adverse effects of oxidized corn gluten meal.

Theaflavin, a bioactive compound extracted from black tea, has been studied for its antioxidant properties in promoting human health, delaying aging, and supporting female reproduction and ovarian function via multiple pathways, including mTOR-mediated autophagy regulation [8]. It also improves glucose metabolism in high-fat diets and streptozotocin-induced diabetic rats [25]. Mechanically, theaflavin binds to egg ovalbumin with an affinity of 104 M-1 [26]. Additionally, it attenuates apoptosis through multiple pathways, including p53 translocation from the cytoplasm to the nucleus, upregulation of GPx-1 in renal ischemia injury [10], suppression of miR-128-3p expression and its inhibitory effect on SIRT1 [27], and protection against oxidative stress-induced apoptosis in PC12 cells [28]. In this study, theaflavin inhibited AOPP-induced ROS, decreased MDA levels, and elevated T-SOD and T-AOC in GCs. Notably, CCK8, a cell activity biomarker, was significantly elevated after theaflavin treatment, prompting an investigation into GC proliferation. Immunostaining revealed increased Ki67 signals in the theaflavin group, suggesting enhanced GC proliferation. Furthermore, theaflavin treatment lowered the percentage of early apoptotic GCs (FITC high, PI low) compared to AOPP treatment alone.

The yellow color of egg yolk results from dietary xanthophyll accumulation [29]. The key factors influencing egg yolk color are the quantity and quality of xanthophyll pigments in feed [30]. Corn gluten meal, a major by-product of corn wet milling, contains (on a dry basis) approximately 60% (*w*/*w*) proteins with highly concentrated carotenoids (200–400 µg/g), the majority of which is xanthophyll [31]. While xanthophyll is relatively stable in its natural state, it becomes sensitive to light, temperature, and chemical exposure during processing [32]. Therefore, heat exposure accelerates xanthophyll degradation [33], reducing egg yolk color.

In our study, egg oxidation was evident, especially in egg white, where DPPH, MDA, and protein carbonyl levels significantly changed in the oxidized gluten meal group compared to the control group. This may be attributed to the degradation of antioxidants caused by high heat during corn gluten meal processing. Heating proteins at high temperatures triggers physicochemical reactions such as peptide structure modifications. Thermal–oxidative decomposition produces monomeric, polymeric, and primary and secondary oxidative compounds, thereby decreasing antioxidant content and affecting protein quality [34,35]. Consequently, consuming oxidized corn gluten meal reduces antioxidant deposition in eggs, thereby reducing their antioxidant capacity. Antioxidative compounds such as egg-derived peptides, tryptophan, tyrosine, vitamin E, and carotenoids contribute to the total antioxidant capacity of egg yolk [36]. Tryptophan and tyrosine, aromatic amino acids known for their high antioxidant activity in egg yolk [37], are particularly affected by heat treatment. Heating above 60 °C significantly reduces aromatic amino acid stability, mainly due to oxidation [38]. This decline in free aromatic amino acids may explain their reduced levels in oxidized gluten meal during heating.

In this study, theaflavin improved the reducing power and decreased MDA levels in egg whites. Previous studies report that tea extracts increase the antioxidant capacity of eggs. Zhou et al. (2021) and Wang et al. (2020) observed that 600 mg/kg of tea polyphenols increased DPPH value and free amino acids (isoleucine, leucine, tyrosine, phenylalanine, tryptophan, lysine, threonine, serine, glutamic acid, valine, and methionine) in egg yolk [39,40]. Wang et al. (2021) observed that 400 mg/kg of TBs improved DPPH value, reducing power, T-AOC, tryptophan, cysteine, methionine, and histidine in egg yolks while reducing MDA content [41]. Our findings align with previous studies, as theaflavin supplementation significantly increased threonine content in egg yolk. Theaflavins, similar to theabrownin and tea polyphenols, may protect aromatic amino acids from oxidative protein-mediated degradation [41]. However, theaflavin does not counteract the loss of free amino acids such as tryptophan and glycine in egg yolk.

## 5. Conclusions

Oxidized corn gluten meal induces oxidative stress in the ovarian GCs of laying hens, triggering apoptosis, impairing egg production, and diminishing egg antioxidant capacity. While theaflavins partially mitigate these adverse effects, future studies are needed to elucidate their molecular interactions with oxidized proteins in regulating antioxidant responses in laying hens and their eggs.

## Figures and Tables

**Table 1:** Ingredients analysis of fresh and oxidized corn protein.

Item (g/kg)	Fresh Protein	Oxidized Protein
Dry matter	908.1	992.7
Crude protein	621.4	628.2
Crude fat	49.8	30.8
Protein carbonyl (nmol/mg of protein)	8.96	42.18
Free sulfhydryl (nmol/mg of protein)	7.92	4.26
Total disulfide and sulfhydryl (nmol/mg of protein)	64.88	51.67

**Table 2:** Basic diet’s ingredients and nutritional composition (fed-basis).

Feed Ingredients	Percentage (%)
Corn	38.30
Soybean meal	13.20
Corn starch	15.00
Corn gluten meal	10.00
Corn oil	2.00
Wheat bran	8.00
Rice bran with hull	0.83
Sodium chloride	0.40
Calcium carbonate	9.70
Calcium hydrophosphate	1.55
Mineral premix ^1^	0.50
Vitamin premix ^2^	0.03
L-Lysine-HCl	0.27
DL-Methionine	0.06
L-Tryptophan	0.06
Choline chloride, 50%	0.10
Nutrient content ^3^	
ME (Mj/kg)	11.35
Crude protein (%)	16.40
Calcium (%)	4.09
Available phosphorus (%)	0.58
Lysine (%)	0.37
Methionine (%)	0.73
Threonine (%)	0.36
Tryptophan (%)	0.54

^1^ Minerals provided the following nutrients per kg of diet: CuSO_4_·5H_2_O, 10 mg; FeSO_4_·H_2_O, 60 mg; ZnSO_4_·H_2_O, 100 mg; MnSO_4_·H_2_O, 100 mg; KI, 1 mg; Na_2_SeO_3_, 0.3 mg. ^2^ Vitamins provided following nutrients per kg of diet: Vitamin A, 8000IU; Vitamin D_3_, 1600IU; Vitamin E, 5IU; Vitamin B_1_, 0.8 mg; Vitamin B_2,_ 2.5 mg; Vitamin B_6_, 1.5 mg; Vitamin B_12_, 0.004 mg; D-pantothenic acid, 2.2 mg; folic acid, 0.25 mg; nicotinic acid, 20 mg; biotin, 0.1 mg. ^3^ Calculated value.

**Table 3:** The effect of theaflavin and oxidized corn gluten meal on the production performance of laying hens (n = 10) ^1^.

Item	−Theaflavin		+Theaflavin		SEM	*p*-Value		
	−OX	+OX	−OX	+OX		Theaflavin	OX	Interaction
Egg production (%)								
1–4 wk	86.58	86.35	87.25	86.72	0.32	0.418	0.554	0.822
5–8 wk	85.49	82.89	87.36	85.17	0.36	0.007	0.002	0.781
1–8 wk	86.19	84.70	87.30	85.96	0.29	0.051	0.021	0.901
ADFI (g/hen)								
1–4 wk	108.34	109.12	107.65	109.67	0.37	0.930	0.070	0.412
5–8 wk	110.89	112.72	109.95	112.58	0.47	0.564	0.022	0.672
1–8 wk	109.88	110.99	109.00	111.13	0.37	0.621	0.036	0.500
FCR (g of feed/g of egg)								
1–4 wk	1.99	2.01	1.97	2.01	0.01	0.634	0.124	0.546
5–8 wk	2.06	2.15	1.99	2.09	0.01	0.002	<0.001	0.900
1–8 wk	2.02	2.08	1.98	2.05	0.01	0.030	<0.001	0.638
Egg weight (g)								
1–4 wk	62.99	63.00	63.00	63.01	0.02	0.899	0.892	0.986
5–8 wk	63.15	63.14	63.14	63.13	0.02	0.770	0.821	0.917
1–8 wk	63.07	63.07	63.07	63.07	0.01	0.970	0.943	0.958
Dirty egg (%)								
1–4 wk	2.29	2.07	2.30	2.14	0.11	0.872	0.390	0.884
5–8 wk	1.79	1.79	1.73	2.08	0.10	0.566	0.394	0.401
1–8 wk	2.04	1.94	2.02	2.11	0.08	0.642	0.965	0.515
Broken egg (%)								
1–4 wk	0.41	0.70	0.66	0.53	0.08	0.808	0.628	0.197
5–8 wk	0.49	0.50	0.79	0.48	0.10	0.466	0.439	0.403
1–8 wk	0.45	0.60	0.72	0.51	0.07	0.524	0.829	0.190

^1^ OX = oxidized corn gluten meal; ADFI = average daily feed intake; FCR = feed conversion rate; SEM = standard error of the mean.

**Table 4:** The effect of theaflavin and oxidized corn gluten meal on antioxidant status of laying hens (n = 10) ^1^.

Item	−Theaflavin		+Theaflavin		SEM	*p*-Value		
	−OX	+OX	−OX	+OX		Theaflavin	OX	Interaction
Liver								
T-AOC (U/mgpro)	1.21	1.09	1.28	1.21	0.04	0.206	0.208	0.765
GSH-*Px* (U/mgpro)	79.41	63.30	81.22	77.92	2.45	0.102	0.055	0.199
SOD (U/mgpro)	218.49	200.90	243.59	234.08	6.17	0.024	0.280	0.746
MDA (nmol/mgpro)	0.70	0.81	0.60	0.83	0.02	0.346	0.001	0.185
Serum								
T-AOC (U/mgpro)	1.38 a	0.79 b	1.01 ab	1.27 a	0.05	0.577	0.129	<0.001
GSH-*Px* (U/mgpro)	ND	ND	ND	ND	ND	ND	ND	ND
SOD (U/mgpro)	412.47	385.83	466.16	399.83	6.34	0.011	0.001	0.126
MDA (nmol/mgpro)	10.80	10.82	10.34	10.75	0.45	0.770	0.810	0.830
Ovary								
T-AOC (U/mgpro)	1.01	0.66	1.16	0.86	0.07	0.231	0.026	0.862
GSH-*Px* (U/mgpro)	79.14	63.64	81.11	67.14	3.01	0.654	0.020	0.900
SOD (U/mgpro)	61.98	56.01	84.20	66.27	3.55	0.028	0.101	0.406
MDA (nmol/mgpro)	1.04	1.36	1.12	1.11	0.05	0.379	0.119	0.101

^1^ Means without a common letter are different, *p* < 0.05. T-AOC = total antioxidant capacity; GSH-*Px* = glutathione peroxidase; SOD = superoxide dismutase; MDA = malondialdehyde; ND = not detectable.

**Table 5:** The effect of theaflavin and oxidized corn gluten meal on the egg quality of laying hens (n = 40) ^1^.

Item	−Theaflavin		+Theaflavin		SEM	*p*-Value		
	−OX	+OX	−OX	+OX		Theaflavin	OX	Interaction
Egg shell strength (kg/cm^3^)	3.95	3.76	3.53	3.69	0.10	0.223	0.936	0.364
Eggshell thickness (mm)	0.43	0.41	0.42	0.42	0.01	0.612	0.485	0.258
Albumen height (mm)	9.65	9.54	9.99	9.69	0.12	0.329	0.410	0.692
Yolk color	9.60	6.61	9.22	6.97	0.09	0.954	<0.001	0.038
Haugh unit	97.31	96.21	98.34	96.66	0.53	0.488	0.193	0.788
L*	74.33	73.01	75.52	74.54	0.54	0.210	0.288	0.875
a*	3.78	4.22	3.57	3.94	0.17	0.475	0.230	0.926
b*	13.89	14.10	13.47	14.39	0.25	0.901	0.259	0.469

^1^ OX = oxidized corn gluten meal; L* = lightness; a* = redness; b* = yellowness. SEM = standard error of the mean.

**Table 6:** The effect of theaflavin and oxidized corn gluten meal on antioxidant capacity of eggs (n = 10) ^1^.

Item	−Theaflavin		+Theaflavin		SEM	*p*-Value		
	−OX	+OX	−OX	+OX		Theaflavin	OX	Interaction
Egg yolk								
DPPH	26.83 a	23.28 b	28.53 a	20.74 b	0.49	0.673	<0.001	0.038
Reducing power	0.27	0.23	0.29	0.24	0.00	0.108	<0.001	0.301
MDA	90.25	93.76	94.09	95.94	1.80	0.410	0.463	0.820
Protein Carbonyl	49.36	59.38	51.26	53.97	1.85	0.638	0.094	0.330
Egg White								
DPPH	42.83	38.49	43.41	39.41	0.61	0.542	0.002	0.890
Reducing power	0.52	0.47	0.55	0.51	0.00	0.001	<0.001	0.675
MDA	8.47	8.96	6.01	7.80	0.19	0.000	<0.001	0.090
Protein Carbonyl	1.24	1.74	0.91	1.67	0.06	0.103	<0.001	0.289

^1^ Means without a common letter are different, *p* < 0.05; OX = oxidized corn gluten meal; DPPH = 2,2-Diphenyl-1-Picrylhydrazyl radical scavenging activity; MDA = malondialdehyde; SEM = standard error of the mean.

**Table 7:** The effect of theaflavin and oxidized corn gluten meal on the free amino acids of egg yolk (n = 10) ^1^.

Item	−Theaflavin		+Theaflavin		SEM	*p*-Value		
	−OX	+OX	−OX	+OX		Theaflavin	OX	Interaction
Aspartic	288.19	239.45	287.01	221.62	9.58	0.623	0.005	0.667
Threonine	363.76	345.70	398.62	389.31	7.44	0.012	0.364	0.771
Serine	444.01	444.39	461.14	443.17	12.23	0.747	0.721	0.710
Glutamic	1110.01	1174.70	1191.38	1129.74	27.40	0.742	0.978	0.257
Glycine	140.89	125.96	150.37	127.46	3.12	0.384	0.004	0.526
Alanine	191.63	223.58	222.32	201.85	4.66	0.634	0.542	0.008
Valine	374.24	376.48	399.58	361.56	6.80	0.704	0.197	0.148
Methionine	166.53	184.20	186.13	170.45	3.04	0.634	0.871	0.009
Isoleucine	316.69	325.87	344.29	334.51	7.35	0.226	0.984	0.523
Leucine	663.27	669.78	684.08	655.48	16.77	0.923	0.744	0.604
Tyrosine	468.81 b	497.16 ab	539.82 a	449.80 b	9.32	0.530	0.107	0.003
Phenylalanine	471.59	420.80	505.19	506.46	16.29	0.076	0.452	0.430
Tryptophan	181.86	156.56	178.64	154.92	3.98	0.762	0.004	0.922
Lysine	585.48	624.38	659.42	594.69	13.54	0.419	0.636	0.064
Histidine	110.93	116.52	106.38	85.80	4.39	0.052	0.399	0.145
Arginine	507.46 ab	507.94 ab	564.22 a	485.63 b	8.58	0.322	0.029	0.027

^1^ Means without a common letter are different, *p* < 0.05. OX = oxidized corn gluten meal; SEM = standard error of the mean.

## Data Availability

The data used to support the findings of this study are available from the corresponding author upon request.

## Abbreviations

The following abbreviations are used in this manuscript:

AOPPs	Advanced oxidation protein products
GCs	Granulosa cells
OX	Oxidized corn gluten meal
CT	The control group
TF	Normal diet group containing 500 mg/kg of theaflavin
CB	The combination group
ADFI	Average daily feed intake
T-AOC	Total antioxidant capacity
GSH-*Px*	Glutathione peroxidase
SOD	Superoxide dismutase
MDA	Malondialdehyde
DPPH	2,2-Diphenyl-1-Picrylhydrazyl radical scavenging activity
